# Differentially expressed *genes* of RNA-seq data are suggested on the intersections of normalization techniques

**DOI:** 10.1016/j.bbrep.2023.101618

**Published:** 2023-12-21

**Authors:** Mohammad Elahimanesh, Mohammad Najafi

**Affiliations:** a Clinical Biochemistry Department, Faculty of Medical Sciences, Iran University of Medical Sciences, Tehran, Iran; b Microbial Biotechnology Research Center, Iran University of Medical Sciences, Tehran, Iran

**Keywords:** Data normalization, RNA-Seq analysis, DEGs

## Abstract

Data normalization is the critical step for the RNA-seq data analysis. Several techniques are suggested for the normalization of transcript reads in the samples. In this study, the differentially expressed *genes* (DEGs) are generated from the TCGA normalized laryngeal cancer data obtained using the TPM, FPKM, and DESeq2 techniques. The results showed that the reports of DEGs were different based on the normalization techniques. We suggested that the DEG intersections obtain the top transcripts from the normalized data in support of the pathway enrichment process.

## Highlights

• Data were normalized with TPM, FPKM and DESeq2 techniques.
• DEGs were estimated through normalization techniques.
• The DEGs intersections identified through statistical parameters.

## 1 Introduction

The omics tools are widely used in high-throughput studies. The transcriptomic studies focused on the changes of transcriptome and regulatory factors in biological samples. The RNA microarray, RNA-seq, and ChIP-seq techniques are applied to identify the RNA copy numbers, transcription elements, and factors. The RNA-seq (RNA sequencing) technique is able to detect the RNA molecules based on the reads mapped for each transcript [1]. The normalization process is necessary in which the raw data are primarily adjusted for the transcriptome analysis. It causes to synchronize the sample data. Some factors, such as sequencing depth, gene length, and RNA composition are applied in the normalization techniques. CPM (Count per million), TPM (Transcript per kilobase million), FPKM/RPKM (Fragment/Reads per kilobase of exon per million), and DESeq2-normalized count (Row count) techniques are suggested to normalize the RNA-seq data. However, the differentially expressed genes (DEGs) are generated from the normalized data, but their quantity and quality depend on the applied normalization techniques. In contrast with the TPM and FPKM/RPKM techniques, DESeq2 compares the data across samples [2,3]. However, data verification and validation need to evaluate the DEGs generated after using each technique.

In this study, the TPM, FPKM, and DESeq2 techniques were applied to normalize the TCGA laryngeal cancer data. Then, the DEGs were generated on the changes in probability (P) and log fold (LOG-FC) values and enriched using the KEGG signaling pathways.

## 2 Methods and materials

### 2.1 Sample data

The RNA-seq data extracted from the TCGA (https://portal.gdc.cancer.gov/) database (Data Category: transcriptome profiling, Data Type: Gene Expression Quantification, Experimental Strategy: Larynx RNA-Seq Data) were belonged to control (n = 11) and laryngeal cancer (n = 117) samples.

### 2.2 Data normalization

Moderate t-statistics were utilized to assess the expression differences between the cancer and normal samples. The TPM, FPKM, and DESeq2 techniques were applied to normalize the raw read data in all the samples (n = 128) [4]. Then, the normalized data were applied for the generation of DEGs.

### 2.3 Differentially expressed genes (DEGs)

**Fig. 1:**
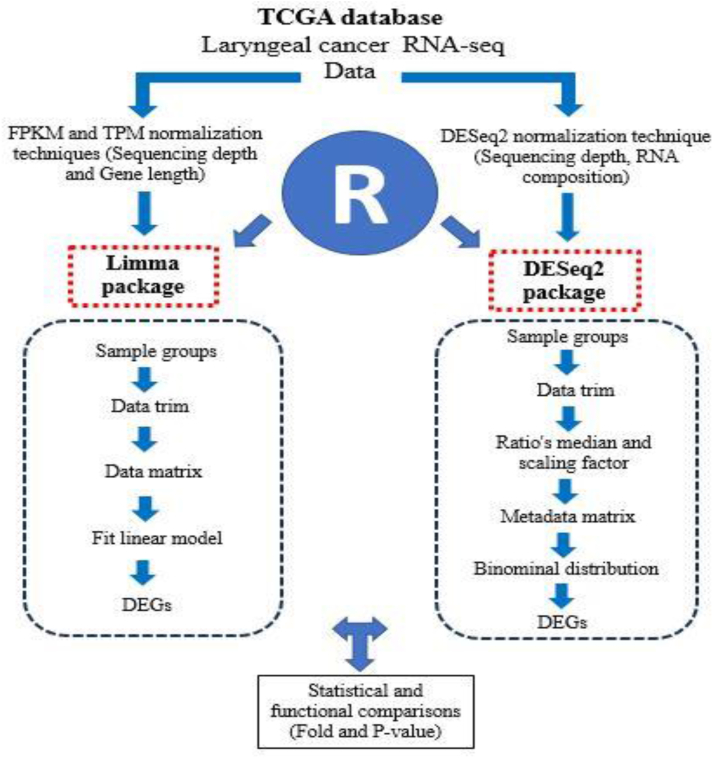
Study procedure. The TCGA laryngeal cancer data were normalized using the TPM, FPKM, and DESeq2 techniques. The DESeq2 package applied the raw counts and Limma package used from the normalized data by TPM and FPKM to generate the DEGs.

The differentially expressed genes (DEGs) were determined using the DESeq2 and Limma packages in the R software. The DESeq2 package applied the raw counts, while the Limma package used normalized data from the TPM and FPKM techniques (Fig. 1). The changes of DEGs were evaluated in the P (0.05–0.00001) and transcript log fold (1-5) values.

### 2.4 Gene enrichment using KEGG signaling pathways

The DEGs were enriched by the KEGG signaling pathways (https://www.genome.jp/kegg/). The most important signaling pathways (the signaling pathways including >100 genes for DEseq2, and >10 genes for TPM and FPKM) were selected and compared on the changes in the P (0.05–0.00001) and transcript log fold (1-5) values between the normalization techniques. The signaling pathways were visualized as nodes, and their sizes were estimated as the number of DEGs found in each pathway.

### 2.5 Statistics

The linear and binominal composition models were applied to generate the DEGs using the Limma and DESeq2 packages. The chi-square (χ^2^) test was used to compare the DEGs obtained through the TPM, FPKM, and DESeq2 techniques. A P value less than 0.05 was proposed to be significant.

## 3 Results

### 3.1 The variance coefficients were highly estimated using the FPKM and DESeq2 techniques

**Fig. 2:**
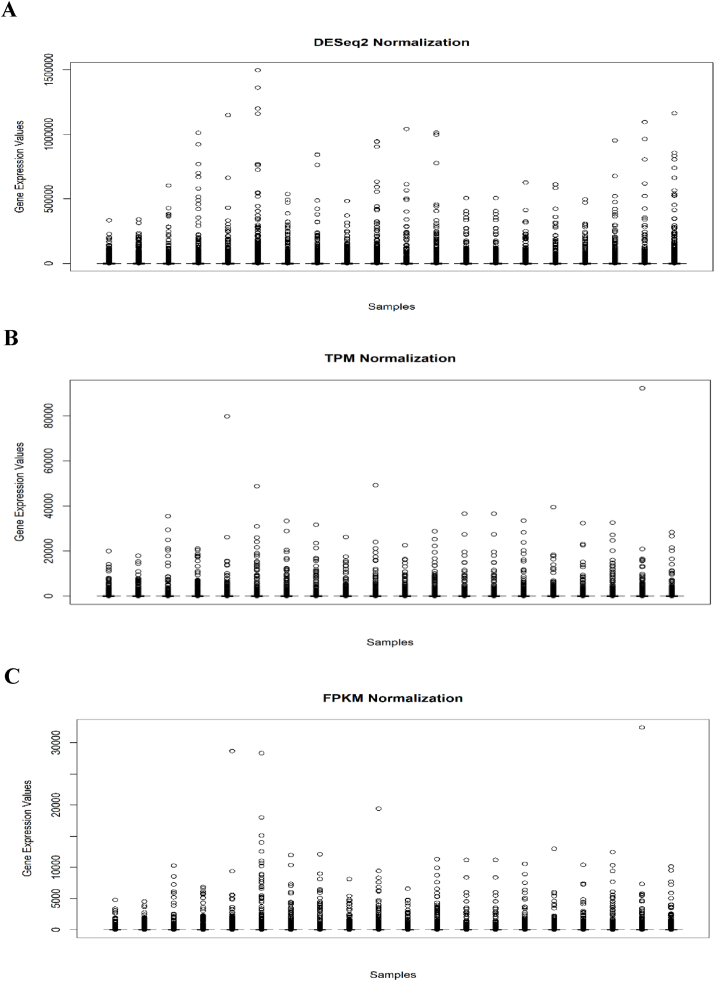
Data normalization. The genes are observed on the normalized read data in several samples. A, DESeq2 technique. B, TPM technique. C, FPKM technique.

The normalization plots were identified using the TPM, FPKM, and DESeq2 techniques (Fig. 2, A, B, and C). The normalized read variance coefficients were highly estimated in the FPKM and DESeq2 techniques (>20 %) so that the data distributions around the means were wider as compared to the TPM technique (<5 %).

### 3.2 The DEGs were highly generated using the DESeq2 package

**Fig. 3:**
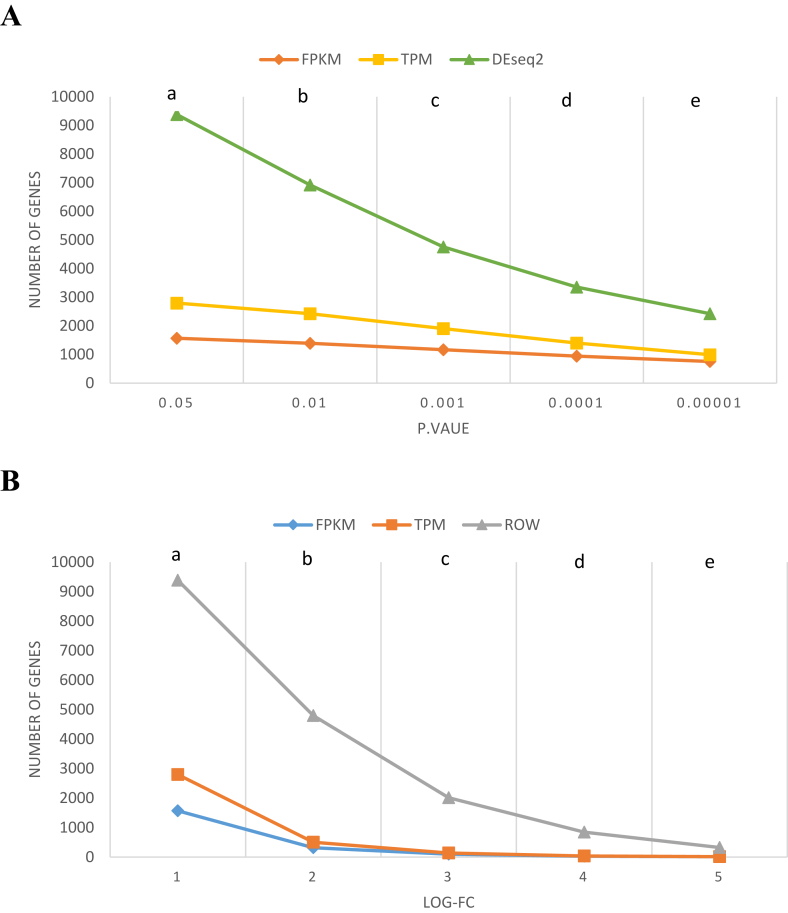
The changes of DEGs obtained from the normalized data using TPM, FPKM and DESeq2 techniques. A, P value changes (a, b, c, d; DESeq2 vs. FPKM and TPM, χ^2^ > 8, p < 0.02: e; DESeq2 vs. FPKM and TPM, χ^2^ = 3.97, p = 0.17). B, Log fold changes (LOG-FC) (a, b, c, d; DESeq2 vs. FPKM and TPM, χ^2^ > 24, p < 5 × 10^−6^: d, e; DESeq2 vs. FPKM and TPM, χ^2^ < 6, p > 0.1).

The Limma and DESeq2 packages determined the DEGs after normalizing the data using TPM, FPKM, and DESeq2 techniques. Then, the DEGs were categorized and compared in the P (Fig. 3, A) and transcript fold (Fig. 3, B) values. The numbers of DEGs were significantly (Chi square >8, P < 0.02) estimated by the DESeq2 technique in the values of P > 0.0001 as compared with TPM and FPKM techniques. However, the DEGs reduced up to 75 % when the P value (0.00001) decreased (Chi square 3.97, P 0.17). The DESeq2 package also significantly identified the DEGs on the log fold values lower than 4 (Chi square >24, P < 5 × 10^−6^). The DEGs were reduced (>98 %) using the DESeq2 technique with an increase in the log fold values (Chi square <6, P > 0.1) as compared to other normalization techniques.

**Fig. 4:**
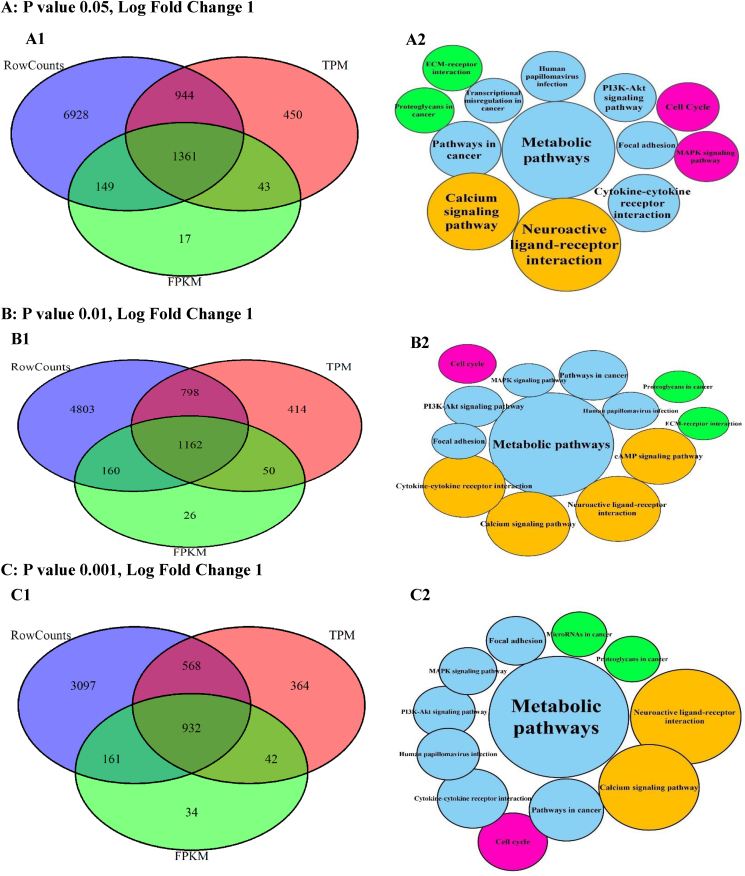

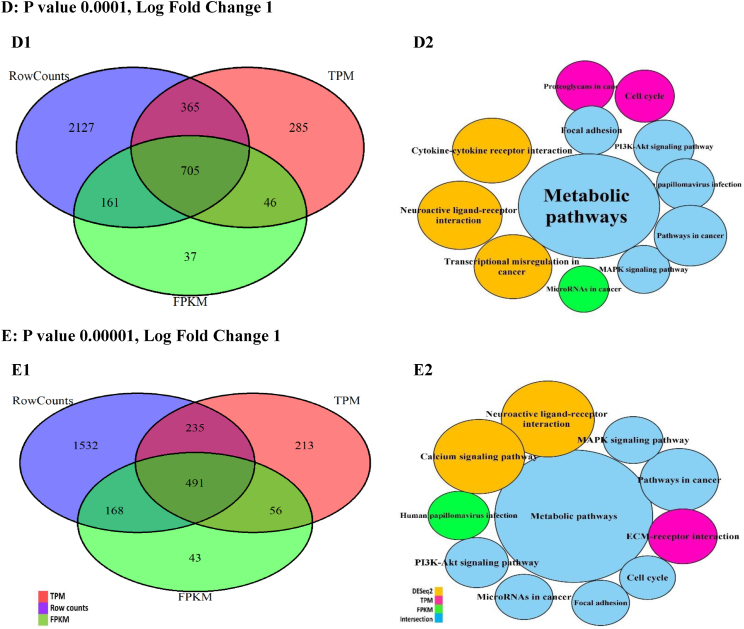
The DEGs plotted by Venn diagrams and enriched by KEGG signaling pathways in the different probability (P) values. The Venn diagrams (A1-E1) showed the intersections of differentially expressed genes (DEGs) generated with the normalized data by the FPKM, TPM, and DESeq2 techniques. The KEGG signaling pathways (Node, A2-E2) and their sizes (DEGs) changed in the P values. However, the important signaling pathways related to DEG intersections preserved on the decrease of P values.

**Fig. 5:**
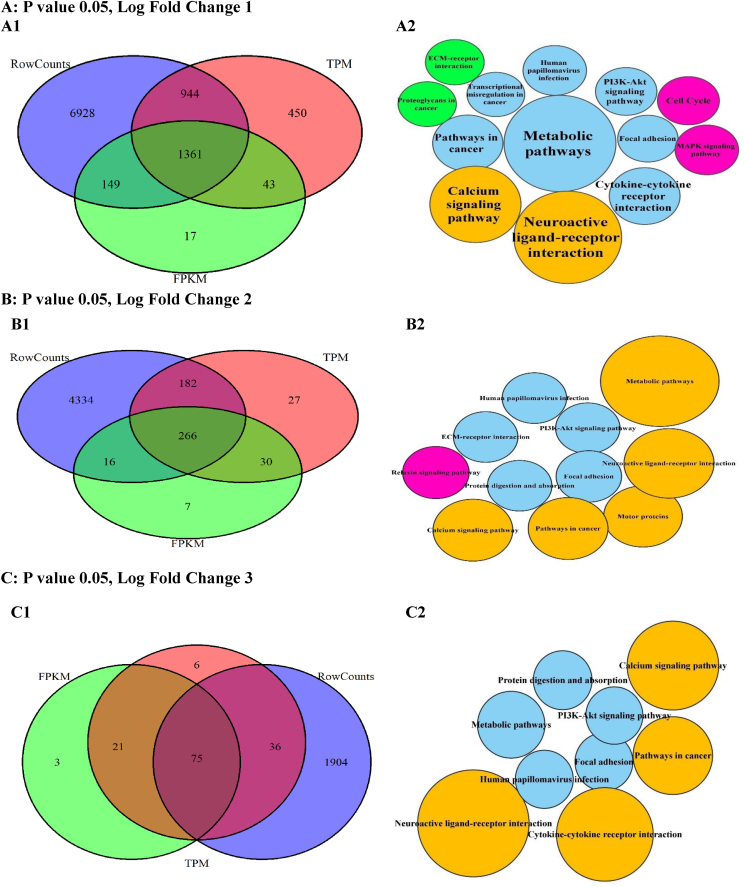

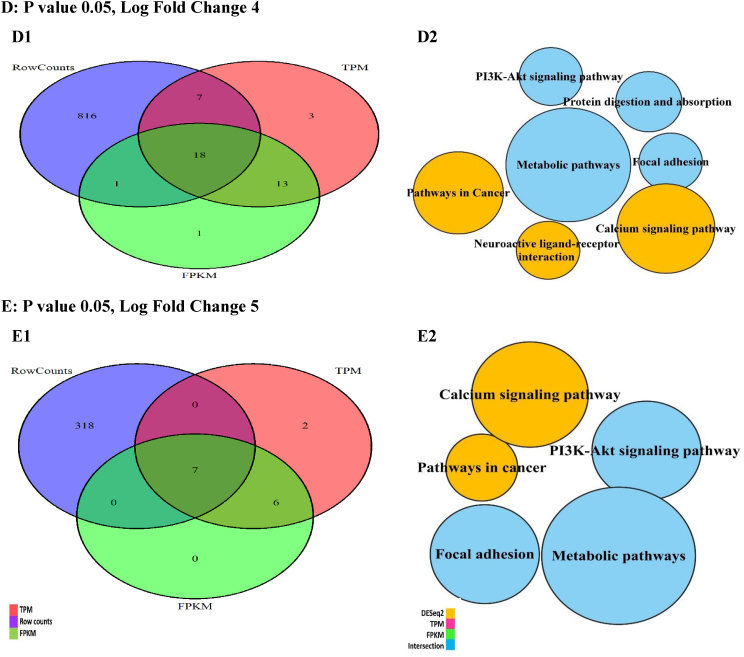
The DEGs plotted by Venn diagrams and enriched by KEGG signaling pathways in the different log fold (LOG-FC) values. The Venn diagrams (A1-E1) showed the intersections of differentially expressed genes (DEGs) generated with the data after the normalization by the FPKM, TPM, and DESeq2 techniques. The KEGG signaling pathways (Node, A2-E2) and their sizes (DEGs) changed in the log fold values.

The DEG intersections among the studied techniques were calculated between 15 and 18 % by changing the P values (Fig. 4, A1-E1), while the DEG unions changed up to 35 %. Moreover, the DEG unions between DESeq2 and TPM were higher as compared with FPKM. These changes were calculated up to 5 % for log fold values (LOG-FC) (Fig. 5, A1-E1).

### 3.3 The important signaling pathways are always shown at the DEG intersections

The DEGs were enriched using the KEGG signaling pathways in the different P and transcript fold values (Fig. 4, A2-E2). Based on the P changes, 13 important signaling pathways were found, so the enrichment of DEG intersection showed 7 signaling pathways with the highest DEG count (P 0.05, fold change 1) directly involved in the cancer. With the decrease in P values, the signaling pathways decreased. However, this reduction included the unrelated signaling pathways while the important signaling pathways (n = 7) were preserved. The enrichment results also showed the quantity and quality of the cancer signaling pathways related to the DEGs reduce with the increase of transcript fold values (Fig. 5, A2-E2).

## 4 Discussion

The normalization process is an essential approach in RNA-seq data analysis. The different parameters, such as the heterogeneity of transcript reads and the gene length can affect the data normalization. While CPM, TPM, and FPKM techniques focus on the normalization of sequencing depth and gene length in the sample data, the DESeq2 technique normalizes the data with the sequencing depth and RNA composition without considering the gene length. Furthermore, the TPM and FPKM used from RPK (Gene read per gene length (kilobase)) and RPM (Gene reads per million) during the normalization process of data in a sample, respectively. The DESeq2 technique uses logarithmic mode on read multiplication between sample genes and the sample median ratios. Based on the above descriptions, DESeq2 technique is recommended to compare samples and the estimation of DEGs in contrast with TPM and FPKM that focused on the data in a sample [4,5]. This study showed the read variance coefficients were larger among the DESeq2 and FPKM techniques as compared to the TPM technique. These results suggested that the wider distribution of normalized data affects the uncertainty and the increased scatter among the sample upregulated and downregulated genes.

Furthermore, the results showed that the DEGs are highly generated through normalized data with the DESeq2 technique. However, the DEGs were affected by the changes in P and LOG-FC values, but there were no significant differences among the studied techniques when the P and LOG-FC values were <0.0001 and > 4, respectively. These results suggested that the high DEG estimations could inversely affect the network parameters and data enrichment. It may be due to the failure to apply the gene length and the high data variance, resulting in decreased sensitivity in the estimation of DEGs. Based on the above results, we suggested the use of DEG intersection between the techniques since it covered all parameters involved in the normalization data, such as sequencing depth, gene length, and RNA composition, and also estimated the lower DEGs (17–18 %). Furthermore, the DEG enrichment by the KEGG signaling pathways supported the DEG intersection, which can find the most important signaling pathways, so that the reduction of P value elevated the specificity of signaling pathways. However, these findings were observed in the LOG-FC changes.

In conclusion, the study results showed that the TPM, FPKM, and DESeq2 techniques normalize the RNA-seq data with the different variance coefficients. Based on these normalized data, the Limma and DESeq2 packages generated the different DEGs by reducing the P values. Furthermore, the results suggested that the use of DEG intersection causes to find the top DEGs so the KEGG pathway enrichment supported these results. Based on the high specificity for the signaling pathways, it was suggested for all transcriptomic studies.

## CRediT authorship contribution statement

**Mohammad Elahimanesh:** Formal analysis, Investigation, Methodology, Software, Visualization, Writing – review & editing. **Mohammad Najafi:** Conceptualization, Data curation, Formal analysis, Investigation, Supervision, Validation, Visualization, Writing – original draft, Writing – review & editing.

## Declaration of competing interest

The authors declare that they have no known competing financial interests or personal relationships that could have appeared to influence the work reported in this paper

## Data Availability

Data will be made available on request.

## Abbreviation

CPM: Count per million
TPM: Transcript per kilobase million
FPKM/RPKM: Fragment/Reads per kilobase of exon per million
LOG-FC: Log fold changes
DEGs: Differentially expressed *genes*
